# Understanding how, why, for whom, and under what circumstances opt-out blood-borne virus testing programmes work to increase test engagement and uptake within prison: a rapid-realist review

**DOI:** 10.1186/s12913-019-3970-z

**Published:** 2019-03-08

**Authors:** Seth Francis-Graham, Nnenna Adaniya Ekeke, Corey Andrew Nelson, Tin Yan Lee, Sulaima El Haj, Tim Rhodes, Cecilia Vindrola, Tim Colbourn, William Rosenberg

**Affiliations:** 1 The National Institute for Health Research: Health Protection Research Unit in Blood Borne and Sexually Transmitted Infections, University College London, London, UK; 2 Royal Free London NHS Foundation Trust, London, UK; 3 The Chinese University of Hong Kong, Hong Kong, China; 4 The National Institute for Health Research Health Protection Research Unit in Blood Borne and Sexually Transmitted Infections, the London School of Hygiene and Tropical Medicine, London, UK; 5 The Institute of Epidemiology & Health, University College London, London, UK; 6 The Institute for Global Health, University College London, London, UK; 7 The Institute for Liver and Digestive Health, Division of Medicine, University College London, London, UK

**Keywords:** Opt-out, Prison, Blood-borne virus, Hepatitis C, Hepatitis B, Human immunodeficiency virus, Testing

## Abstract

**Background:**

Prisons represent a unique opportunity to diagnose blood-borne viruses. Opt-out testing is receiving increasing interest, as a result of mounting evidence to suggest that the manner in which a test offer is delivered, affects test uptake. Although the effectiveness of opt-out testing within the prison setting has been established, robust explanations are required for the variation in outcomes reported.

**Methods:**

Rapid-realist review methodology was used to synthesise the literature on prison-based opt-out testing. The review was carried out in three phases. Phase one: An expert panel provided literature relevant to the implementation of opt-out testing within the English prison estate. Unstructured searches were also conducted to identify other social programmes where “opt-out” had been used to increase uptake. Phase two: a systematic search of six peer-review and five grey literature databases was carried out to identify empirical data on opt-out testing within the prison setting. Phase three: Additional non-exhaustive searches were carried out to identify literature that reinforced emergent concepts. The development of programme theory took place with each iteration and was validated in consultation with stakeholders.

**Results:**

Programme theory was constructed for two outcomes: the proportion of intake offered a test and the proportion offered that accepted testing. The proportion of intake offered testing was influenced by the timing of the test offer, which was often delayed due to barriers to prisoner access. The decision to accept testing was influenced by concerns about confidentiality, fear of a positive diagnosis, a prisoner’s personal interpretation of risk, discomfort with invasive procedures, trust in healthcare, and the fidelity of the opt-out offer.

**Conclusions:**

This review identified important implementation considerations that moderate the effectiveness of opt-out testing programmes. It also highlighted a lack of appreciation for the theoretical underpinnings of opt-out programmes and tension around how to implement testing in a manner that adheres to both default theory and informed consent. It is anticipated that results will be used to inform the design and implementation of subsequent versions of these programmes, as well as catalyse further in-depth analysis into their operation within the unique context of prison.

**Review registration:**

CRD42017068342.

**Electronic supplementary material:**

The online version of this article (10.1186/s12913-019-3970-z) contains supplementary material, which is available to authorized users.

## Background

Hepatitis C (HCV), hepatitis B (HBV), and the human immunodeficiency virus (HIV) are blood-borne viruses (BBVs), which cause significant morbidity and mortality globally. Biomedical innovation has provided effective treatment options for infection with these BBVs [1]. However, in light of new global targets aimed at the elimination of viral hepatitis by 2030 [2] and 90–90-90 HIV targets for 2020, the need for effective case-detection strategies is becoming increasingly acute [1, 3].

Opt-out testing for BBVs has been suggested as a method of case detection, in light of positive results from other opt-out health programmes, such as opt-out organ donation and opt-out antenatal HIV testing [4–6]. It involves a subtle shift in the way the test offer is delivered [7]. Unlike opt-in testing, opt-out does not require a person to expressly agree to undergo a test, instead they are notified that testing will be performed unless they explicitly decline [8]. Opt-out therefore represents a more paternalistic approach to eliciting consent [9].

Opt-out BBV testing has begun to filter into routine medical practice, often targeted at high-risk communities [7]. Prisons represent a particularly high-risk environment, with the criminalisation of drug use and the association between drug dependency and crime, elevating the prevalence of BBV infection [10, 11]. In response, the Centers for Disease Control and Prevention has recommended opt-out HIV testing within US prisons [11] and in 2014, phased implementation of an opt-out testing strategy for HIV, HCV, and HBV, developed by Public Health England (PHE), the National Health Service England (NHSE), and Her Majesty’s Prison and Probation Service (HMPPS), began throughout the English prison estate [5]. Dried-blood spot testing (DBST) has been recommended for use in the English strategy, as it requires minimal training and can be easier to perform for those with a history of injecting drugs [12].

Systematic review evidence suggests that opt-out programmes can increase test uptake, when compared with opt-in, whilst avoiding many of the ethical debates that surround mandatory testing [13]. However, there exists considerable variation in the way opt-out BBV test programmes are implemented and consequently the outcomes produced [5, 13].

Although some explanations have been offered for these different outcomes [7, 13], there remains little robust theory to help better implement and run opt-out testing programmes within prison. This review sought to remedy this, by explaining how, why, for whom, and under what conditions opt-out programmes testing for HIV, HCV, and HBV within prison are likely to be effective [14, 15]. Results were targeted at the English prison estate, although it was hoped that they would also serve as a general guide for other programmes in other contexts.

## Methods

This study utilised a rapid-realist review (RRR) approach [16, 17]. The PROSPERO reference for the protocol is: CRD42017068342. RAMESES reporting standards were used to guide this study [17].

Realist methodology is increasingly being used to explore the “black box” of complex health programmes, with the aim of constructing an explanatory framework for how, why, and when they work [18]. Applied to secondary research, realist reviews have emerged as robust ways of developing these insights [15, 16, 19]. However, realist reviews are expansive and consequently resource intensive [16]. Out of necessity, RRRs emerged as an alternative, time-responsive, approach suited to small and emerging bodies of literature, or as a first step of a multi-phase project [16, 20, 21].

RRRs develop programme theory, an explanation for how a programme brings about intended and unintended changes to a social phenomenon within a given context [18, 22, 23]. RRRs develop this theory by describing the interaction between the programme’s context, the generative forces stimulated by the intervention (mechanisms), and outcomes. Programme theory is therefore often expressed using the Context-Mechanism-Outcome (CMO) heuristic of realist evaluation [23]. As the constituents of these CMOs can be interpreted in different ways, the review team clarified their shared understanding early in the review process (see glossary of terms in Table 1) [18, 22].

**Table 1:** Glossary of terms

Term	Definition
Realist review	A theory-driven approach to synthesising secondary research (including quantitative, qualitative, or mixed methods research). It aims to develop an explanatory model for how a programme (or different programmes) bring about a recorded change, why, for whom, and under what circumstances. It does this by developing realist programme theory, expressed as Context + Mechanism (Resource/Response) = Outcome.
Rapid-realist review (RRR)	An adapted form of realist review, which provides a truncated method for the development of realist programme theory, whilst preserving the core elements of realist methodology. It relies more explicitly on stakeholders to focus and expedite the review process.
Programme theory	An explanation for how a programme works. Realist reviews and RRRs attempt to develop and test programme theory.
Provisional programme theory	A hypothesised explanation for how a programme is expected to work. Realist reviews and RRR usually start by developing a provisional programme theory to be tested using the literature.
Refined programme theory	The product of a realist review or RRR. An explanation for how a programme works in practice, based on empirical data identified by the review.
Context-mechanism-outcome (CMO) configuration	A heuristic used in realist reviews or RRRs to structure an explanation for how a programme, or part of a programme, works. CMO configurations act as the building blocks for programme theory.
Context	Covers the programme context and the broader contextual backdrop that the programme is situated within, which modify the expression of mechanisms [22].
Mechanism	The “underlying entities, processes or social structures, which operate in particular contexts to generate outcomes of interest” [18]. This review focused on the individual reasoning and preference construction, which occurred in response to resources implemented by the opt-out testing programme [79].
Outcome	Both the intended and unintended consequences of the opt-out testing programme. Outcomes can be proximal, intermediate, or final [22].
Nudge Theory	Nudge is a “substantive theory” (i.e. a theory that exists within a discipline, which can be used to help understand the way a programme works). Utilised in the fields of behavioural science and economics, it describes various quirks of human behaviour and decision-making and suggests ways these can be used to encourage certain actions.
Default Effect	A theory within Nudge, which suggests that for any choice or action, there is a tendency for the majority of individuals to stick with the default option.

### Review process

The RRR followed the broad steps detailed by Saul et al. (2013). The review team began by securing support from the London BBV Core Steering Group, a commissioning body tasked with overseeing BBV service provision (including opt-out testing) for the London prison estate. The group was comprised of stakeholders from NHSE, PHE, HMPPS, the Hepatitis C Trust, and representatives from the different healthcare providers at each London prison, and therefore acted as both an expert panel and reference group (providing knowledge on current practice). Through collaboration with the Steering Group, a series of research questions were developed [16]:

1. *“What are the key outcomes of public health interest, from opt-out testing within a prison context?”*
2. *“What are the generative forces, catalysed by opt-out testing programmes for BBVs within prison, that produce these outcomes?”*
3. *“How does the physical and social context of different prisons, influence the expression of these generative forces?”*
4. *“What is recommended to improve these public health outcomes?”*

**Fig. 1:**
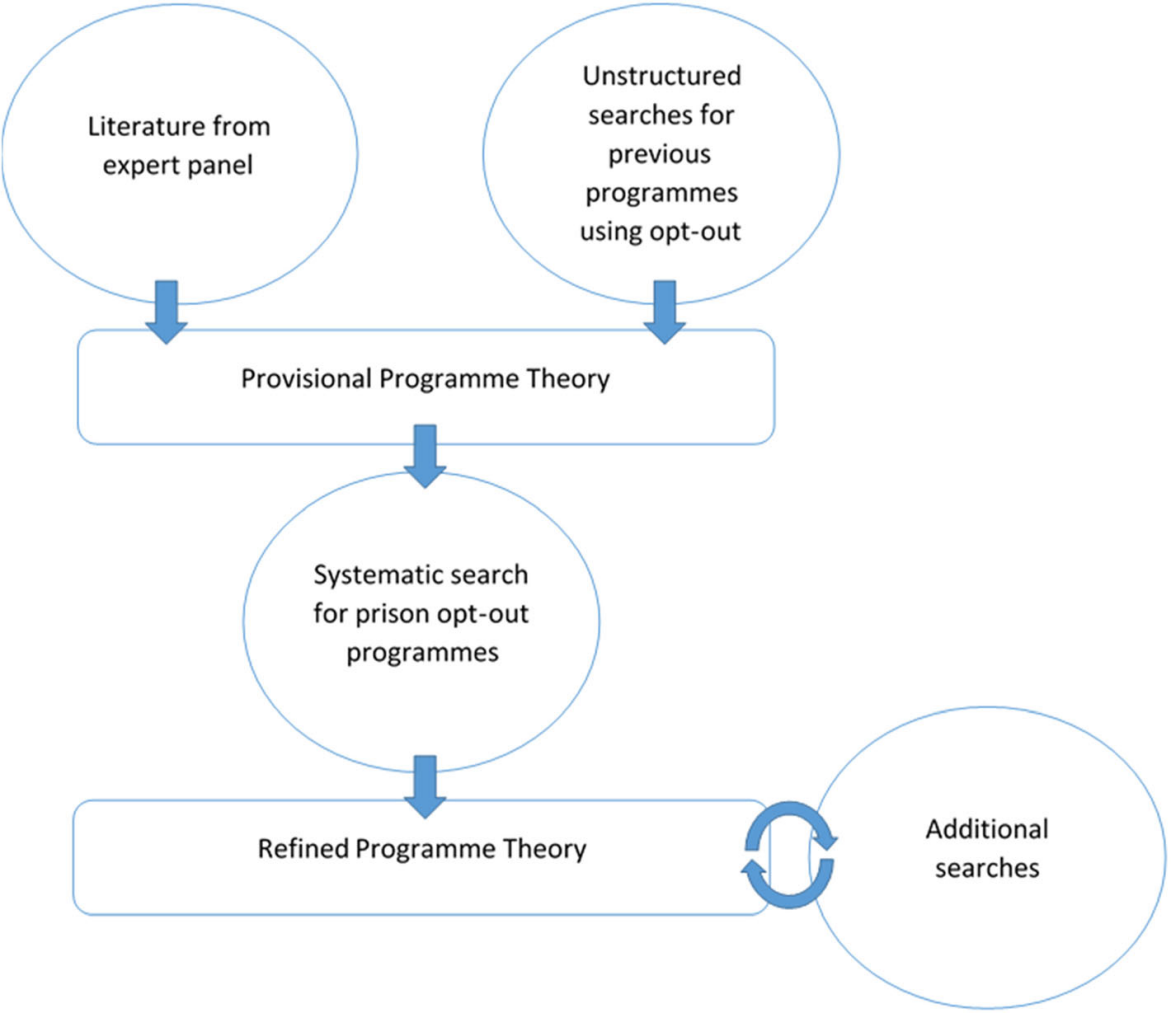
Review process used to develop a refined programme theory for opt-out BBV testing within prison

To answer these questions, iterative searching was used to construct, refine, and reinforce a programme theory for opt-out BBV testing using the literature (Fig. 1) [15, 16]. As the search and analysis process varied between each phase, it is described sequentially. All search results were handled using MENDELY bibliographic software.

### Phase one

In phase one, the review team narrowed the scope of the review and developed a provisional programme theory [15, 16]. To do this, the Steering Group supplied the research team with documents used in the development of the English opt-out test programme [16]. In collaboration with the Steering Group, a generic process for opt-out testing was developed and two outcomes of public health interest identified.

Provisional programme theory was then constructed around these two outcomes. Documents sent by the Steering Group were supplemented with literature identified via a series of unstructured searches, carried out on Google/scholar and MEDLINE for previous programmes that used opt-out to enhance uptake. Phase one articles did not undergo a formal process of eligibility review or quality appraisal, as they were only used to develop the framework that structured subsequent review iterations [15].

### Phase two

With the provisional programme theory developed, the review team then conducted a systematic search for empirical data to refine that theory. A structured search algorithm was developed for bibliographic databases and piloted in MEDLINE, in consultation with a database expert based at the Royal Free London NHS Foundation Trust’s Medical Library. Search terms focused on opt-out testing within a prison context and were informed by Rumble’s et al. (2015) search strategy for a systematic review of routine test methods for BBVs in prison (Table 2) [13].

**Table 2:** Population, location, exposure table, summarising search terms used during the systematic search of online databases

Population	Location	Exposure
• Prisoner* • Offender* • Convict* • Detainee* • Inmate* • Incarcerated	• Prison* • Gaol* • Jail* • Penal institution* • Correction* or penal or remand* or detention or custody) adj2 (centre or department or facility* or system*) • Penitent*	• Mass screen* • (Mandatory or systematic or routine or compulsory or obligatory) adj (test* or screen* or diagnos* or identif* or assess) • Opt-out • Opt* out

Word root searching (denoted using the symbol “*”) was frequently used to find variant forms of a single word

The search did not specify testing for HCV, HBV, or HIV, meaning articles discussing cousin interventions, which nonetheless could be useful for theory refinement, would be identified [15]. MEDLINE, PsycInfo, EMBASE, Scopus, CINHAL+, and ASSIA were all searched using the systematic algorithm (amended as required for each database) in June 2017 (Additional file 1). A search of five grey literature databases (ProQuest Dissertations and Theses Global, DART-Europe-E-Theses Portal, Open Grey, Google Scholar, and “.GOV”) was also carried out in June 2017 [18].

Search results underwent a formal process of eligibility assessment and quality appraisal. Each stage was conducted independently by at least two authors, with disagreements resolved by discussion with a third author. Citations had titles and abstracts reviewed against the following criteria: “Does the citation indicate a prison context?”; “Does the citation indicate testing for a physical disease?”; and “Does the citation discuss physical disease in a population not an individual?”

Any citations that failed to answer “yes” to these questions were excluded. Citations then had their full-text downloaded and were assessed against three dimensions of relevance: “Provides information related to mechanisms triggered by opt-out testing”; “Provides information on outcomes of opt-out testing”; “Provides contextual information related to opt-out testing within prisons”.

Articles that did not provide information on one of these dimensions were excluded. A traffic light system was then used to highlight how many dimensions were covered by each article (red articles covered one dimension, orange two, and green three). Each reviewer also assigned articles a subjective score from 1 to 10, to indicate how useful they believed it would be in the analysis. An average of the two reviewers’ scores was taken and assigned alongside the colour, enhancing transparency, and allowing for some prioritisation of articles. Articles designated red and with a low score (≤4), were reviewed by authors again and their inclusion discussed.

The Mixed Methods Quality Appraisal Tool [24] was used for quality assessment of primary research and the Critical Appraisal Skills Programme: Systematic Review Checklist for systematic reviews. The unit of analysis was the contributing evidence, although an overall quality score was assigned [15]. If a piece of information within an article was deemed low quality, it was excluded, but other pieces of data of acceptable quality were retained from the article [15, 19]. As grey literature and literature review articles did not undergo quality assessment, data on context and mechanism were included if it was supported by, or consistent with, data from other empirical articles, but quantitative outcomes were not used.

Data from articles were annotated and coded as either context, mechanism, or outcome. This evidence was then grouped into a realist matrix, allowing for theming across the matrix [25]. Data was synthesised with the provisional programme theory developed during phase one, via a process of adjudication and amalgamation, producing a refined list of CMO configurations (CMOc) [15]. These were discussed in data meetings with authors and validated during meetings with the expert panel and reference group [16]. One author also observed staff training and opt-out testing conducted within two English prisons, further helping validate CMOcs.

### Phase three

Following phase two, a refined programme theory had begun to take shape. However, to further reinforce the CMOcs developed, a series of purposive unstructured searches were undertaken on MEDLINE and Google/scholar [18, 26]. Searches primarily focused on acquiring qualitative and theoretical articles, discussing testing for BBVs in prison and non-prison settings. It was not an exhaustive process, but aimed to purposefully draw together a diverse range of literature, which was then used to reinforce the theoretical “backbone” of the refined programme theory [26].

Phase three articles did not undergo a formal process of eligibility assessment, but were assessed for quality using the Mixed Methods Quality Appraisal Tool [24]. They did not contribute evidence to outcomes, but were included as they reinforced aspects of context and mechanism [26].

## Results

**Fig. 2:**
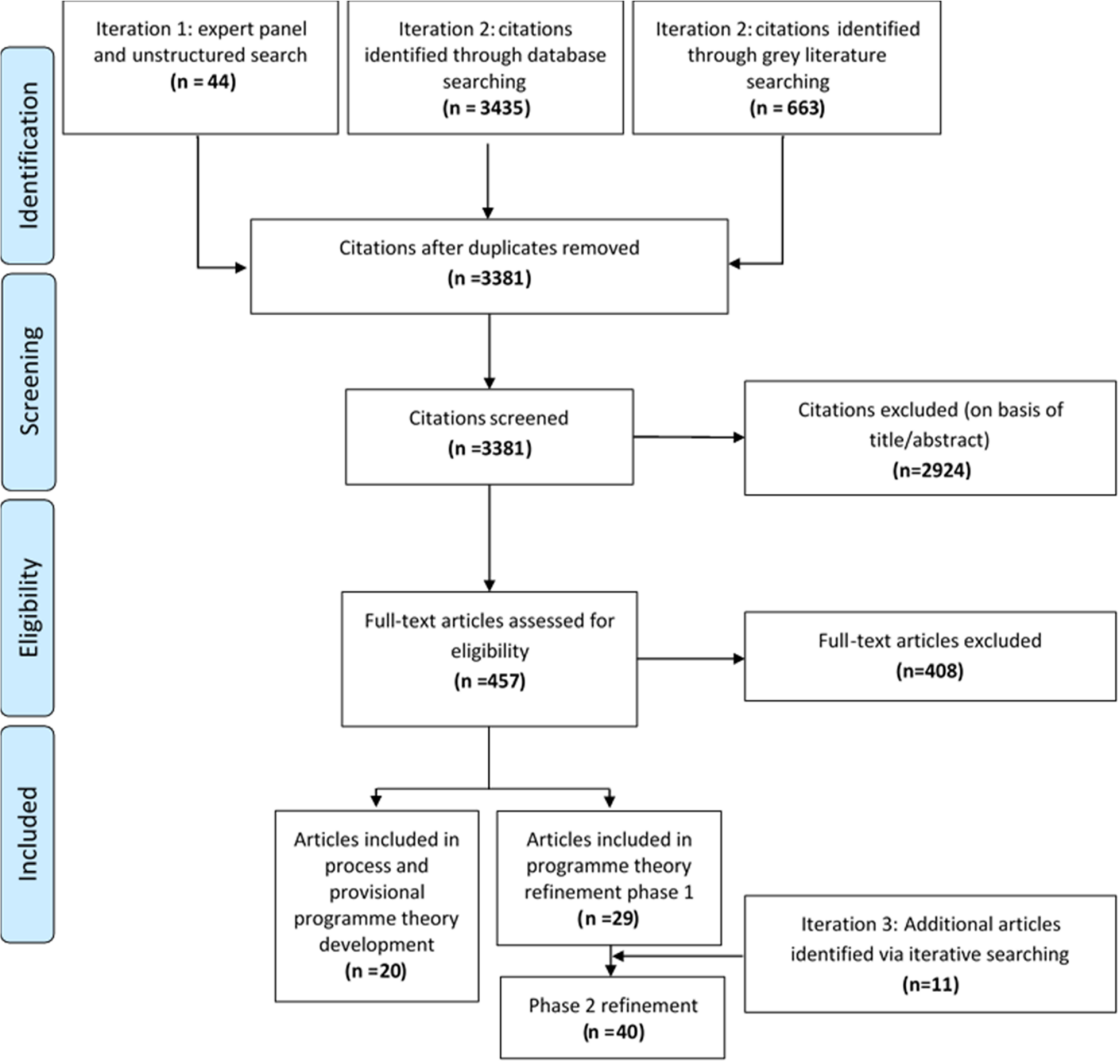
Flow diagram detailing the search results of the rapid-realist review. Diagram design guided by recommendations made by the PRISMA Group (2009) [80]

The expert panel supplied the research team with 26 documents and 18 articles were identified via unstructured searching (Fig. 2). A further 3435 citations were identified via database searching and 663 through grey literature searching. After duplicates were removed, 3381 titles and abstracts were screened, and 457 articles remained for full-text review. 11 documents from the expert panel and 9 articles from the unstructured search (Additional file 2) were used in framework, process, and provisional programme theory development. 29 empirical articles (Table 3) were used in programme theory refinement. These were supplemented with a further 11 articles identified through purposive unstructured searching (Additional file 3).

**Table 3:** Characteristics of phase 2 studies, summarising first author/year, country, prison, disease, study design, method of data collection, aims of research, relevance, and quality assessment score (acceptable: ^b^, good: ^c^, excellent: ^d^

First author, year	Country	Prison	Disease	Study design	Data collection	Aims	Dimensions of relevance	Strength of relevance	Quality score
Kavasery, 2009 [43]	U.S.	Urban men’s jail – New Haven Connecticut	HIV	Prospective controlled trial	Quantitative data capture	Determine the optimal timing of opt-out HIV testing for newly incarcerated jail detainees.	Red Orange Green	9	^d^
Beckwith, 2011 [41]	U.S.	Rhode Island Jail	HIV	Mixed-methods: sequential explanatory	Routine data and interviews/FGD	Introduce rapid opt-out HIV testing to Rhode Island Jail.	Red Orange Green	8	^d^
Public Health England, 2015 [5]	U.K.	Mixture of phase 1 “pathfinder” prisons	HIV, HCV, and HBV	Project evaluation	Questionnaire	Evaluation of opt-out testing for blood borne viruses, implemented throughout pilot English prisons.	Red Orange	4	N/A
Elkington, 2016 [59]	U.S.	Mixed	HIV	Literature review	Systematic search	To review the effectiveness of HIV testing and linkage programmes and review barriers and facilitators to these programmes in the correctional setting.	Red Orange	4	N/A
Rosen, 2016 [52]	U.S.	North Carolina	HIV	Before and after study	Routine data	Assess the impact of routine opt-out testing in terms of case detection.	Red	5	^d^
Rice, 2011 [44]	U.S.	Wayne County Jail	HIV	Thesis	Multiple	Design, implement, and evaluate a jail-based HIV testing program.	Red Orange Green	10	N/A
Spaulding, 2015 [38]	U.S.	Fulton County Jail	HIV	Mixed-methods: sequential explanatory	Routine data and questionnaire	To establish a rapid opt-out HIV testing program, led by the jail-based nursing team.	Red Orange Green	6	^c^
Lucas, 2016 [39]	U.S.	Eight prison reception centres (California)	HIV	Quantitative descriptive evaluation	Routine data	Conduct an evaluation of routine HIV services, implemented throughout California.	Red	4	^c^
Rosen, 2007 [63]	U.S.	8 intake prisons in North Carolina	HIV	Thesis	Routine data	Evaluation of a large southern state opt-out HIV testing programme.	Red Orange Green	5	N/A
Schoenbachler, 2016 [55]	U.S.	Durham County Jail, Florence Detention, Orangeburg Jail, Marion Jail and Darlington Jail	HCV	Quantitative descriptive evaluation	Routine data	Evaluate an HCV testing and linkage-to-care post release program among detainees of small-to-medium sized jails.	Red Orange	5	^b^
Grinstead, 2003 [64]	U.S.	Mixed	HIV, HCV, HBV, and other sexually transmitted infections	Qualitative exploration	Interviews	Explore providers’ experiences regarding HIV, hepatitis, and other sexually transmitted infection testing services within prison.	Red Orange	7	^c^
Centres for Disease Control, 2011 [49]	U.S.	Washington State Department of Corrections (12 male facilities)	HIV	Quantitative descriptive evaluation	Routine data	To assess the rate of testing under three different testing strategies: on-request, routine opt-in, and routine opt-out.	Red Orange Green	5	^c^
Centres for Disease Control, 2009 [11]	U.S.	N/A	HIV	Opt-out testing programme guidance	N/A	To guide the implementation of opt-out HIV testing in the correctional setting by highlighting suggested common components and tenants of such a testing programme.	Red Orange	6	N/A
Peter, 2009 [45]	U.S.	Orleans Parish Prison, Jefferson Parish Correctional Centre	HIV	Thesis	Routine data	Look at the effectiveness of opt-out and opt-in approaches to HIV testing in jail populations.	Red Orange	7	N/A
Muessig, 2016 [57]	U.S.	North Carolina State Prison System	HIV	Qualitative	Interviews – 76 incarcerated men and women	Exploring issues of HIV stigma within an opt-out testing programme.	Red Orange Green	10	^c^
Walker, 2005 [54]	U.S.	N/A	HIV	Letter(s)	N/A	Discusses the ethical concerns surrounding routine opt-out HIV testing within the prison setting.	Red	4	N/A
Beckwith, 2010 [67]	U.S.	N/A	HIV	Literature review	Search	Provide a review of the current state of delivering HIV testing, prevention, treatment and transition services to incarcerated populations.	Red Orange	4	N/A
Rosen, 2015 [8]	U.S.	North Carolina State Prison System	HIV	Quantitative cross-sectional survey	Quantitative survey and routine data	To explore prisoners understanding of the voluntary nature of routine opt-out testing.	Red Orange	8	^c^
Grodensky, 2016 [48]	U.S.	North Carolina Prison System	HIV	Quantitative cross-sectional survey	Quantitative survey and routine data	Estimate the proportion unaware of being tested and the proportion of people tested who did not want a test.	Red Orange Green	9	^c^
Cole, 2014 [46]	U.S.	Cook County Jail	*Chlamydia trachomatis* & *Neisseria gonorrhoeae*	Retrospective analysis	Routine data	Evaluate the impact of opt-out testing on rates of testing and diagnosis of infection among incarcerated women, assess the proportion of infections successfully treated, and evaluate factors associated with receipt of treatment.	Red Orange Green	8	^c^
Public Health England, 2016 [70]	U.K.	Pentonville Prison	HIV, HBV, and HCV	Pilot evaluation	Routine data	Report results from provisional data analysis for the pilot blood-borne virus care pathway trialled within Pentonville prison.	Red Orange	5	N/A
Jack, 2016 [51]	U.K.	East Midlands Category B male prison	HCV	Qualitative phenomenology	Interviews (prison officers)	To explore the views of prison officers about people in prison being tested and treated for HCV.	Red Orange	6	^d^
Beckwith, 2012 [53]	U.S.	Baltimore Department of Corrections, Philadelphia Prison System, District of Columbia Department of Corrections	HIV	Quantitative descriptive evaluation	Routine data	To assess the feasibility of implementing large scale rapid and routine opt-out testing programmes for HIV in large urban jails.	Red Orange Green	6	^d^
Centres for Disease Control, 2013 [37]	U.S.	Fulton County Jail	HIV	Quantitative descriptive evaluation	Routine Data	Evaluate a routine opt-out testing programme in a large county jail.	Red Orange Green	5	^c^
Centre for Disease Control, 2010 [77]	U.S.	Rhode Island Jail	HIV	Quantitative descriptive evaluation	Routine Data	Review of Rhode Island Jail’s testing records.	Red Orange	4	^c^
Kavasery, 2009 [42]	U.S.	York Correctional Institution, Connecticut	HIV	Prospective controlled trial	Quantitative data capture	Evaluate the optimal time to conduct routine opt-out HIV testing of newly incarcerated jail inmates in a manner that maximises the number of individuals capable of consenting and wiling to be tested.	Red Orange Green	9	^d^
Newlan, 2016 [40]	Indonesia	Banceuy Prison	HIV, HBV, and HCV	Natural experiment	Routine data	To compare the efficacy of two different testing strategies (routine or targeted).	Red Orange Green	5	^b^
Rumble, 2015 [13]	Mixed	Mixed	HIV, HBV, and HCV	Systematic review	Systematic literature search	Describe components of routine HIV, HBV, and HCV testing policies in prisons and quantify testing acceptance, coverage, result notification, and diagnosis.	Red Orange Green	7	^d^
Gagnon, 2012 [61]	N/A	N/A	HIV	Literature review	Search	Provide a sociological critique of mandatory testing in light of other testing approaches, including opt-out.	Red Orange	7	N/A

### Process theory and contextual framework

**Fig. 3:**
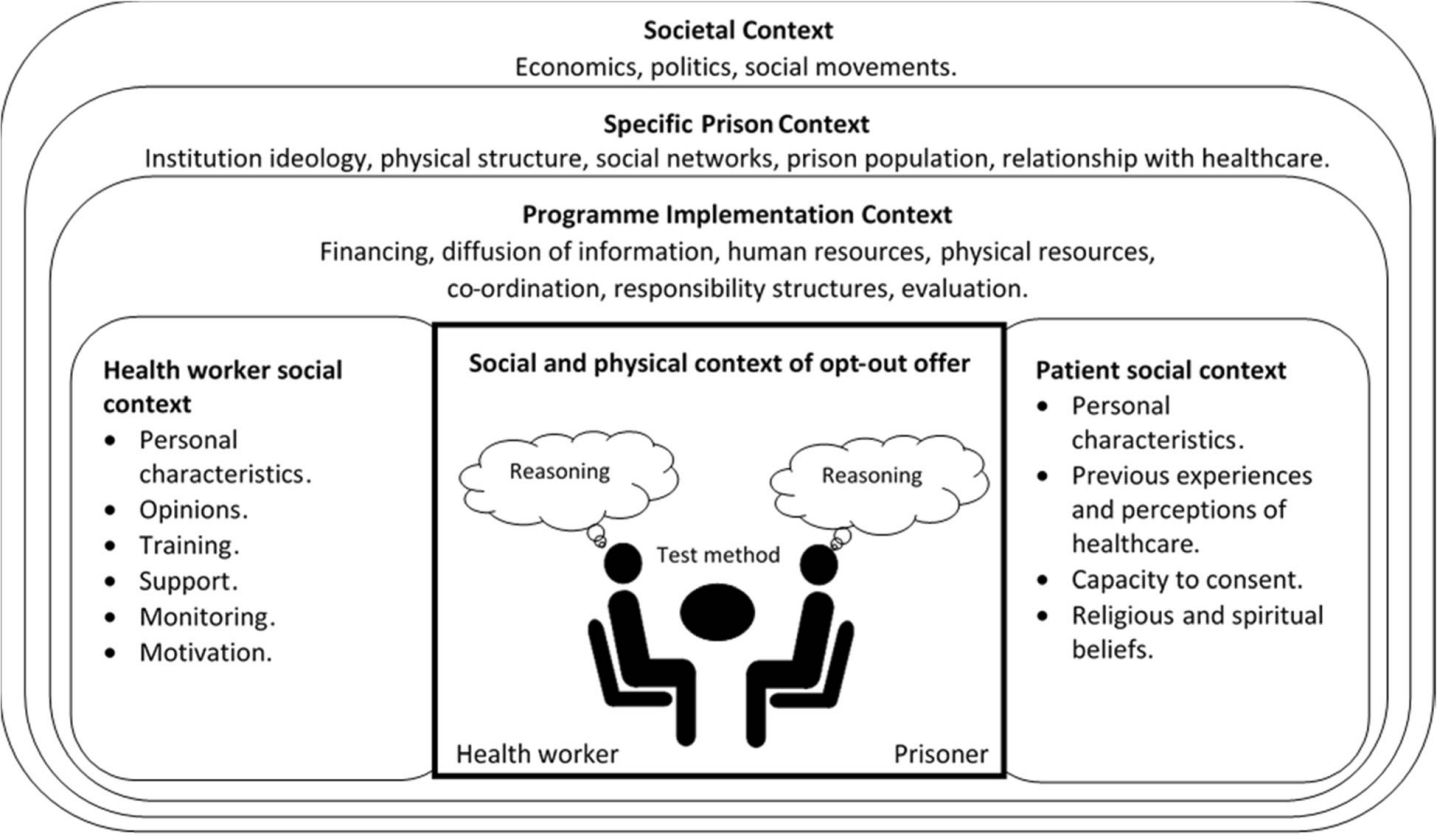
The different spheres of context, influencing the reasoning process of two key actors involved in the opt-out test offer

Reports identified by the expert panel were used to develop a generic process for opt-out testing (Additional file 4). A range of potential outcomes of public health interest were identified. The research team focused on the proportion of intake offered a test and the proportion offered that accepted testing, as these outcomes were highlighted by the Steering Group as key targets for the opt-out intervention [16, 27]. A framework was also developed, to aid the research team conceptualise the sphere of contextual influences that could affect these outcomes (Fig. 3).

### Provisional programme theory

**Fig. 4:**
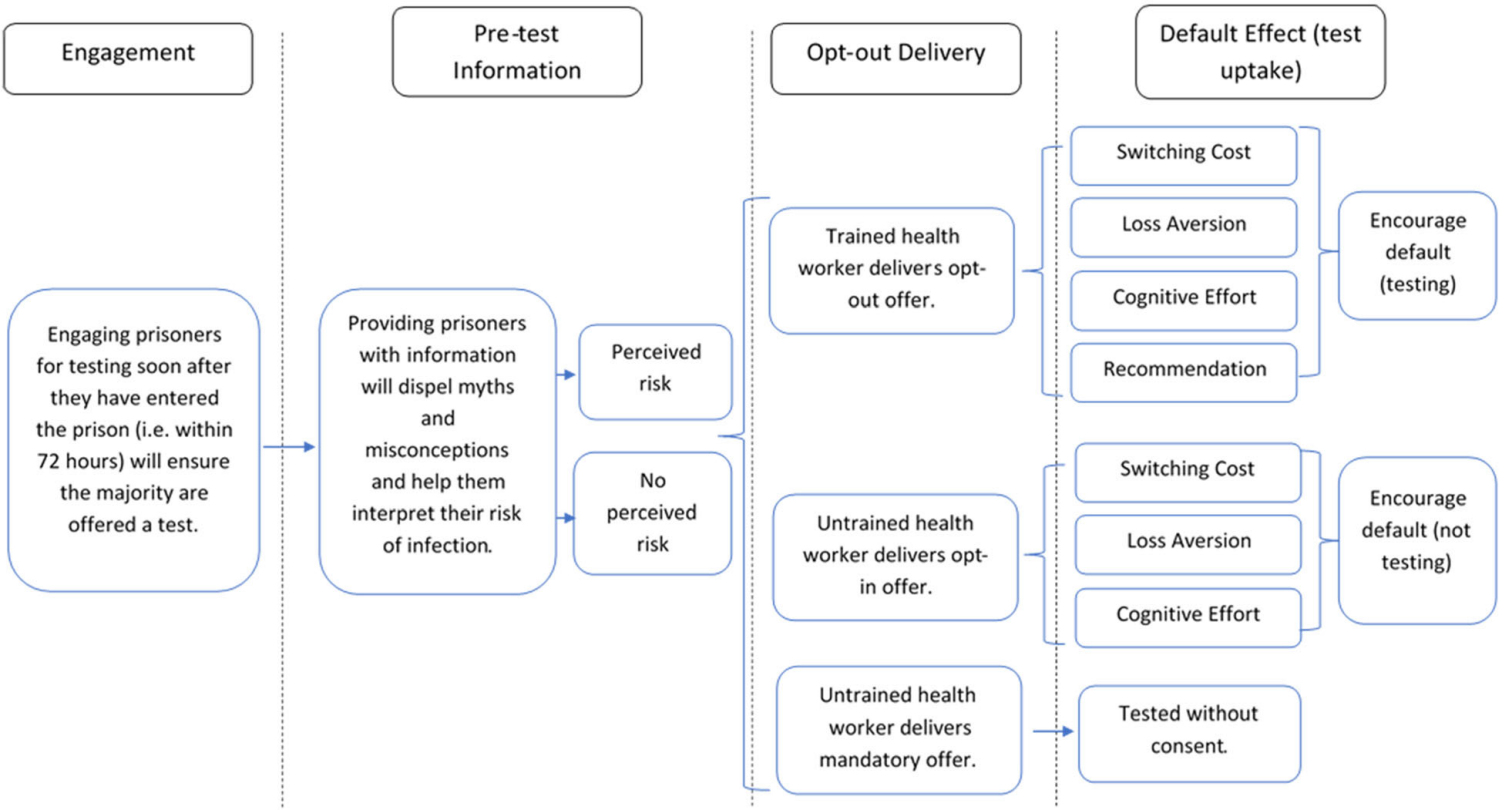
Provisional programme theory developed using articles acquired from phase one

Using data from phase one, provisional programme theory was constructed around the two outcomes selected (Fig. 4).

It was hypothesised that the timing of the test offer would influence the proportion of prisoners offered a test [28–30].

In terms of test uptake, educational information covering transmission risk, symptoms, and the importance of testing was anticipated to play a priming role during the test offer, helping prisoners more accurately interpret their risk of infection and assess costs and benefits of testing [31].

The way testing was offered was also expected to influence uptake. Offering testing in an opt-out manner is not be the norm for health workers, therefore training was considered essential to ensure test offers were opt-out in practice (Fig. 4) [32].

Finally, the unstructured searches carried out during phase one, found literature that suggested the Default Effect, a component of Nudge Theory, underpinned opt-out [18, 25]. The Default Effect suggests that for any choice or action, there is a tendency for individuals to stick with the default option [4]. By aligning the default option of a BBV testing programme with the public health objective (prisoner takes a test), opt-out was hypothesised to encourage test uptake in a variety of ways [4, 33, 34]:

1. **Switching cost:** Individuals incur a cost (e.g. having to justify decision or fill out a form) when opting-out of testing. If this cost exceeds the benefit of opting out, then it is irrational for the individual to do so [35].
2. **Loss aversion:** Individuals tend to weight losses more heavily against equivalent gains. By making testing the default option, loss of benefits provided by testing are weighted more heavily against potential gains of not testing [36].
3. **Cognitive effort:** Making an active decision requires cognitive effort. By making testing the default option, opt-out testing exploits individuals bias not to expend this effort, encouraging those who do not exhibit a strong preference to test [35].
4. **Recommendation:** Making testing the default option, acts as an implicit or inferred recommendation to test [4, 36].

### Refined programme theory

6 CMOcs for the proportion offered testing and 7 for test uptake are presented. Additional file 5 contains the full list of CMOcs developed from the RRR.

Under each CMOc, background information is provided. The configuration is then presented in italics, with components explicitly highlighted: C = context, MR = mechanism resource, MRE = mechanism reasoning response and O = outcome. Exemplifying data, where available, is then presented, providing the reader access to empirical evidence that contributed to theory development and refinement. As reported in other realist reviews, empirical evidence rarely presented a clear description of all three constituents, making abductive reasoning critical to ensure complete CMOc articulation.

### Proportion offered testing

There was significant variation in the proportion offered testing between different prison-based opt-out programmes, ranging from 13 to 100% [13, 37–43]. Failure to offer testing was an implementation issue, operating at various conceptual levels of prison context (Fig. 3).

### CMOc 1: Delayed test offer

The timing of the test offer was a salient factor affecting the proportion offered testing [5, 11, 13, 39, 42–47]. When prisoners first arrive, all new intake usually undergo a first night health check. Seven studies reported opt-out testing conducted during this process [8, 37, 38, 45, 46, 48]. Seven other articles reported testing taking place anywhere between 3 and 14 days after first reception, often during a secondary health check [39, 40, 44, 49–52]. Testing at a secondary clinic often occurred because of a perceived lack of time during the first night or because the first night health check was reserved for dealing with urgent healthcare problems that required immediate intervention [39, 40, 44, 49–52].

*In a prison that has a rapid population turn-over (C), a programme mandated delay in engaging intake with an opt-out test offer (MR) reduces the proportion of intake offered a test (O), as some individuals have already been released or transferred (C).*

This was exemplified during Beckwith’s et al. (2012) evaluation of rapid-HIV testing within three urban jails. A 3–4 day delay in the Baltimore Department of Corrections, resulted in a 13% test offer proportion compared to 100 and 89% respectively in the Philadelphia Prison System and District of Columbia Department of Corrections, which offered testing during a first night health check [53].

### CMOc 2: Early testing and capacity to consent

The desirability of first night testing was tempered by the need for informed consent [11, 37, 42, 43, 47, 54].

*A higher proportion of prisoner’s lack capacity to consent on the first night (*e.g. *undergoing substance abuse withdrawal) (C). As opt-out testing requires informed consent (C), health workers that identify this lack of capacity (MR) and view it as important (MRE) will not offer testing (O).*

This was highlighted in two prospective control trials conducted in US jails, which found 10–11% of new intake were not medically competent to be tested immediately upon entrance, limiting the utility of first night testing [42, 43]. This dropped to 0–4% when testing took place 1–7 days after first reception [42, 43].

### CMOc 3: Prioritisation of security and prison processes

Prison officers were important gatekeepers, as they tended to dictate prisoner movement within the prison environment [44–46, 51, 55].

*Prison officers have a challenging role, particularly when budget cuts have strained the workforce (C). Opt-out testing often requires prison officers to collect prisoners, bring them to clinic, and supervise them (MR). Officers prioritise security and prison processes over escorting and monitoring prisoners at clinic (MRE), meaning prisoners frequently do not arrive, or are not allowed to be at the clinic, to be offered testing (O).*

This process of prioritisation was demonstrated in quotes from qualitative work with prison officers: “*the issue with the health should be considered, if its’ not life threatening.*. *.then security should be the priority*” [51]. This was also highlighted by health workers: *“I think you can’t get away from the fact that we’re entirely dependent on prison officers to deliver healthcare services.*. *.We’ve lost, since I’ve been here, 25% of prison officers.*. *.Who would have thought that ‘do not attends’ are a massive problem in prison?”* [30].

### CMOc 4: Provider capacity to run clinics

Stretched health teams meant that testing clinics could not be properly run, resulting in prisoners being missed [5, 11, 47, 56].

*Prisons are a demanding place to work (high burden of mental illness, physical morbidity, and regular medical emergencies) (C) and budget deficits result in health staff cuts (C). These working conditions reduce the capacity of health staff (MR), forcing them to prioritise certain activities (MRE), such as dealing with urgent conditions or emergencies, resulting in testing clinics being delayed or cancelled and prisoners not offered a test (O).*

Insufficient staffing was most frequently reported in response to the question “*what other barriers did you encounter when trying to complete an HIV test.*. *.at intake?”*, delivered to providers in a New York City Jail [47].

### CMOc 5: Refusal to attend clinic

Prisoner agency also acted as a barrier to offering testing under certain conditions [13, 44, 45].

*When testing is conducted concurrently with other prison activities (C), attendance at clinic becomes an opportunity cost for the prisoner (MR). If health is a lower priority, relative to the other activity (MRE), the prisoner will not attend clinic (O).*

Programme stakeholders reported prisoners refusing to come to clinic because they were sleeping, watching TV, playing sport, and attending gym [13, 44, 45].

### CMOc 6: Rebooking prisoners

When prisoners failed to attend clinic or when clinics were cancelled, health workers were required to rapidly rebook them for testing [5, 11, 47, 56].

*Budget deficits have led to health staff cuts (C). Stretched health workers (C) that are required to re-book prisoners (MR), prioritise medical emergencies and conducting other tasks that require immediate attention (MRE), further delaying the test offer (O). Overworked health staff (C) may also forget to rebook a prisoner (MRE), delaying the realisation of the test offer (O)*.

*In high-turnover prison settings (C), a failure to rapidly rebook a prisoner (MR), reduces the proportion of people offered testing (O), as individuals may be released or transferred by the time they are rebooked (C).*

### Test uptake

The proportion of prisoners that accept a test under opt-out varied from 22 to 98% [13, 37–43]. There was a notable lack of switching costs, with most programmes simply requiring prisoners to verbally opt-out [8, 41, 48, 53]. Several costs and gains associated with opt-out BBV testing within a prison context were also identified. These were activated and modified depending on the presence of certain programme resources.

### CMOc 1: Confidentiality and stigma (loss aversion)

Confidentiality was a key resource for opt-out testing programmes, as the enclosed environment of prison amplifies fear of infectious disease amongst prisoners and staff [51, 57, 58].

*BBVs are stigmatised within the prison context (C). Maintenance of confidentiality (MR) is therefore crucial, as prisoners will feel safe (MRE) to share personal information (O). If a prisoner distrusts prison healthcare’s ability to maintain confidentiality (MR), they may fear stigma (MRE), encouraging opt-out (O).*

Officers view infectious prisoners as a personal risk and may attempt to elicit confidential information from health staff [51, 58]. The close contact between staff and prisoners also means information can be spread, both within and between staff and prisoner groups: *“Would I tell somebody else, a close friend, if I knew they were in contact? Possibly yeah?” (prison officer)* [51]. Breaches in auditory and visual confidentiality can also occur when conducting testing, as a result of the confined environment, security requirements, and the increasing reliance on prisoners for the maintenance of the prison environment [39, 47, 51, 57–61].

### CMOc 2: Coping with a positive diagnosis (loss aversion)

Incarceration is stressful and the potential diagnosis of an infectious disease, often perceived as terminal, can be daunting [13, 38, 43, 54, 59, 60, 62–64].

*BBVs are a situational concern for many people within prison (C). The provision of supportive information (*e.g. *treatment options, dispelling myths around prognosis, and psychosocial support) (MR), reassures a prisoner about coping if they test positive (MRE), encouraging test uptake (O).*

*Failure to provide supportive information (MR) can leave people in prison feeling unable to cope with the perceived burden associated with a positive diagnosis (treatment, stigma, psychological distress, lifestyle changes) (MRE), encouraging opt-out (O).*

This was captured in quotes from health staff: *“Some clients will refuse to take the test out of fear of a positive result”* [38] and prisoners: *“Er, I don’t know really, [pause] er, I don’t really know, I mean, I think like I say, I think people are just frightened ye na. People are frightened to get the test ye na, thinking that it could be a killer not knowing what, not knowing what it actually is, what it actually does to you, I mean?”* [60].

### CMOc 3: Fear of an invasive procedure (loss aversion)

A fear of needles was frequently highlighted as a justification for opt-out [12, 13, 40–42, 57, 64–66].

*A proportion of prisoner’s fear needles (C). When testing is conducted using a venous sample method (MR), prisoners that are uncomfortable with the method of blood acquisition (MRE) may opt-out (O).*

This was captured in quotes from health workers: *“.*. *. I would say nine out of ten people say ‘I hate needles’ and tense up and freak out, and some people are really upset by it”* and “*They were definitely more compliant with it [oral testing]; they’re more willing to get it done as opposed to getting their blood drawn*” [41]. Less invasive sample measures, such as DBST or oral testing, may therefore help to minimise discomfort as a barrier to testing.

### CMOc 4: Institutional recommendations and trust (loss aversion/recommendation)

Making testing the default option acts as an implicit recommendation to test. Positive encouragement from staff can also reinforce this message [44, 45].

*Recommendations to test in circumstances of trust (C) provide an institutional social pressure (MR) that encourages an individual to comply with the perceived positive action (MRE), encouraging test uptake (O). However, institutional distrust is prevalent in prison (C). Institutional social pressure (MR) can be perceived as a coercive process of surveillance, triggering resistance from the individual (MRE) and encouraging opt-out (O)* [59, 64, 67].

### CMOc 5: Personal interpretation of risk (loss aversion)

Educational information on BBVs was an important resource for opt-out programmes [13, 40, 47, 48, 57, 60, 63, 68].

*Misconceptions around BBVs are common amongst prisoners (C). Prisoners that have been informed about modes of transmission and symptoms of the disease (MR) are empowered (MRE) to accurately interpret their risk of infection (O).*

*For prisoners that self-identify as “at risk” (C), testing can be an opportunity to confirm serostatus (MR), allowing the individual to either confront infection (MRE) or be reassured by a negative result (MRE), encouraging test uptake (O).*

In the absence of supportive resources, people in prison that see themselves as “at risk” may feel unable to cope and instead opt-out (see CMOc 2 in this section) [13, 38, 60].

*Prisoners that interpret themselves as low risk (C), but that face no other barriers to testing (MR), may still seek reassurance (MRE), encouraging test uptake (O). Prisoners that face other barriers to test uptake (*e.g. *fears around confidentiality or dislike of test method) (MR), may view testing as an unnecessary burden (MRE) and opt-out of testing (O).*

A range of articles reported issues with the delivery of educational information, with this stage of testing often being truncated [13, 42, 43, 45–47, 54, 60]. In the absence of educational information, people in prison often inaccurately interpreted themselves as low risk, due to a lack of symptoms, or because they had tested previously [13, 37, 38, 40, 42–44, 46, 50].

### CMOc 6: Defaults and capacity to consent (cognitive effort)

New prisoners often suffer from substance withdrawal, have untreated mental health conditions, are physically exhausted, and emotionally overwhelmed [11, 42, 43]. By making testing the default option and offering testing soon after prison entrance, individuals may be tested without understanding what it is they are testing for [8, 69].

*New prisoners frequently lack capacity to provide informed consent (C). If the health worker fails to identify this and proceeds with an opt-out test offer (MR), these individuals may misunderstand what is taking place (MRE) or be unable to make an active decision to opt-out (MRE), instead appearing to comply with testing (O).*

Grodensky et al. (2016) found that out of 871 patients undergoing an opt-out HIV test, 103 were not aware of being tested, 94 did not want to be tested, and 30 were not aware they were tested and did not want a test.

### CMOc 7: Opt-out fidelity

The distinction between eliciting consent in an opt-in, opt-out, or mandatory manner is nuanced and difficult to operationalise in practice [5, 8, 48, 70]. The review highlighted variation in the delivery of an opt-out test, which may partially account for variation in test uptake.

*If programme implementers misinterpret how to deliver an opt-out test (C), training and scripts provided to health workers (MR) will encourage them to comply (MRE) with the delivery of either an opt-in (O) or mandatory (O) test offer.*

*An opt-out test offer is not the norm (C). When health workers have little training, and no standard script (MR), the meaning of opt-out may be misinterpreted (MRE) resulting in either opt-in (O) or mandatory (O) test offers. The way testing is offered, when there is no standard script (MR), can also morph with each encounter, with rapport (C), situational distractions (C) and fatigue (C) all potentially influencing test delivery (O).*

A survey conducted by Rosen et al. (2015) as part of an opt-out testing programme that had a 95% test uptake [8, 48], found that less than 40% of prisoners identified testing as voluntary, which was attributed to an ambiguous consent process and widespread failure of nurses to mention a prisoner’s right to decline the test [8].

## Discussion

60 articles were synthesised to provide CMOcs explaining how, why, for whom, and under what conditions opt-out programmes for HIV, HCV, and HBV might generate a high proportion of test offers and test uptake. The unstructured search conducted during phase one, identified a number of articles highlighting that the Default Effect underpins “opt-out”. It was notable that no documents supplied by the expert panel during phase one and none of the articles from phase two mentioned Nudge Theory or the Default Effect as a consideration in the development or subsequent evaluation of opt-out BBV testing within prison [35]. It appears that these concepts, which underpin the intervention, have been widely forgotten, as “opt-out” is reproduced by stakeholders in different contexts [71].

### Offered a test

Implementation factors were found to significantly limit the proportion of prison intake offered testing [8, 37, 38, 45, 46, 48]. Programmes that implement testing early can increase the proportion of people offered a test (particularly within high-turnover prisons), but may be inhibited by greater volumes of individuals unable to provide informed consent [42, 43]. Further work is required to determine whether the timing of the test offer has an impact on test uptake [42, 43]. Where possible, prisons with a short average incarceration length should look to conduct testing on the first night, during second reception, and at any appropriate subsequent clinics, in order to balance risk of release and capacity to consent [42, 43, 56].

**Fig. 5:**
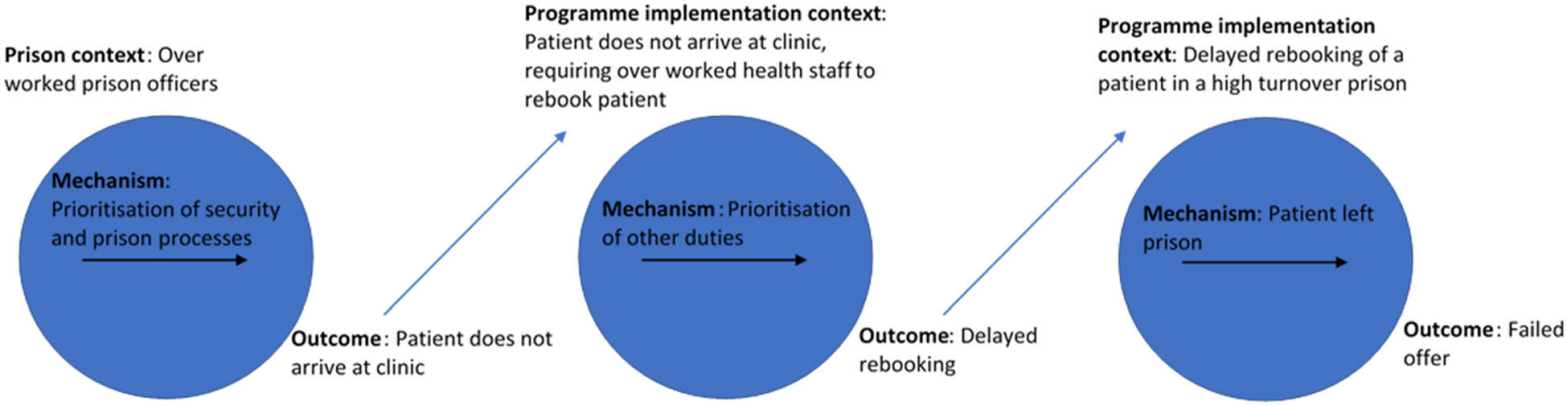
Example causal chain, with various intermediary outcomes that lead to the final outcome of public health interest. Each intermediary outcome forms the context of a subsequent programme theory. This casual chain operates between two spheres of context, detailed in Fig. 3 (the “Specific prison Context” and the “Programme implementation context”)

It was noted that testing was also subject to a range of barriers, which limited provider access to prisoners. These barriers further delayed the realisation of the test offer and operated in causal chains [72], with several intermediate outcomes leading to the final outcome of a failed test offer (example in Fig. 5). Even when programmes specify an appropriate period within which the test offer should occur, given the average incarceration length of their population, it is likely that a proportion of prisoners will be engaged much later, all the time risking release.

The operational capacity of prison officers and health workers were reoccurring contextual features within these causal chains [5, 11, 47, 56]. Historic de-valuation of prisoner well-being is often enacted through budget cuts to the prison estate, reducing the operational capacity of the staff [73]. Greater emphasis should be placed on prisoner well-being via appropriate funding of the prison estate, both out of public health and ethical necessity [74].

More immediate options for opt-out programmes struggling with engagement could include: complimentary sub-interventions to foster collaboration between health workers and prison officers, educational events in prison to encourage prioritisation of the programme, and incentivising clinic attendance for prisoners (e.g. by making it a compulsory pre-cursor for gym attendance) [44–46, 51, 55].

### Test uptake

Varying numbers of people offered a test were found to opt-out [13, 37–43]. The lack of compelling switching costs, implemented by many opt-out programmes reviewed, may be explained by the absence of Nudge Theory in programme conception. Much of the power of opt-out strategies, when used in sectors like marketing, comes from “sleight of hand” tactics (e.g. using miniature font) [4, 34, 35]. Those implementing healthcare programmes do not have the luxury of such tactics and therefore need to optimise their use of defaults, whilst working within the ethical paradigm of informed consent [4, 34]. Programmes struggling with test uptake could therefore consider piloting minor switching costs (e.g. an opt-out justification form that prisoners are asked to complete [44]), although caution should be exercised to ensure that this does not become coercive.

The review also highlighted ethical considerations, related to the exploitation of individual bias not to expend cognitive effort under opt-out [4, 35]. Given the vulnerability of the population group, providers need to be vigilant of capacity to consent when offering testing, ensuring those that do not make an active decision about testing, do so from a lack of preference as opposed to inability as a result of substance withdrawal or mental illness [35, 42, 43].

**Fig. 6:**
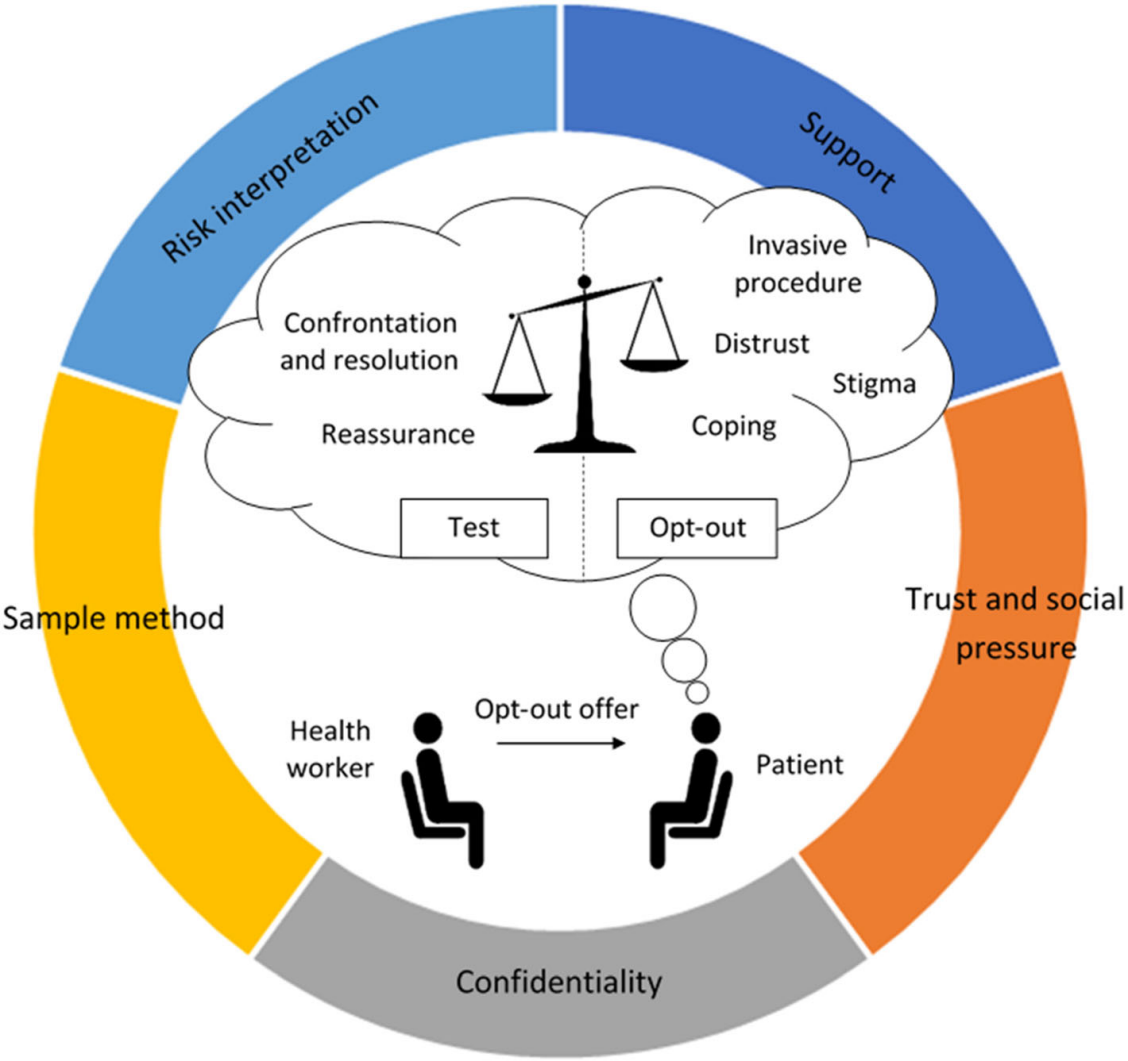
Costs and benefits related to blood-borne virus testing within a prison context. Salient contextual resources, which influence the realisation of these costs and benefits are depicted surrounding the person’s decision making. Loss aversion suggests that the scales should be initially balanced in favour of testing under opt-out

Prisoners’ decision to accept testing or opt-out was however found to be influenced by a range of costs and benefits related to testing for BBVs. Although loss aversion suggests that this weighting should be in favour of testing, significant costs were identified, which provided a strong counterbalancing force (Fig. 6) [34, 35].

Resources such as confidentiality, education, trust, psychosocial support, and less invasive sample methods were found to be important at mitigating testing costs and encouraging test uptake (Fig. 6). These resources were frequently built into the opt-out programmes reviewed [5, 11, 27]. For programmes that experienced issues with the implementation of one or several of these resources, further research is required to unpick why these different programme resources were unsuccessfully realised within different prison contexts.

Finally, refined theory highlighted challenges to the fidelity of opt-out, which stemmed from the conceptualisation of the offer by programme implementers, misinterpretation by those delivering the test offer, and/or due to contextual pressures [5, 8, 48, 70]. The need to acquire consent can naturally lead to asking a person if they would “like to test” [42, 43], failing to fully adhere with principles of opt-out and potentially limiting uptake [7, 13]. However, not informing a prisoner that they have the right to decline, given the punitive context, borders on a mandatory approach [75, 76] and raises ethical questions if prisoner’s interpret it as such [8, 48].

Further work is required to determine what constitutes an opt-out offer and how adherence to opt-out can be ensured in practice. However, a written set of words for health workers to use when offering testing, could help to standardise the process [5, 7, 8, 48, 70]. A set of words conceived by NHSE and PHE commissioners, but not implemented during the English service reconfiguration, were synthesised with findings from this review to develop a recommended set of words that were then shared with the London BBV Core Steering Group (Additional file 6).

### Limitations

Much of the empirical data came from opt-out HIV testing conducted within US prisons, potentially limiting the applicability of refined theories to the English prison context, where HIV, HCV, and HBV are tested for together, commonly using DBST. However, validation with English stakeholders, as well as observation of opt-out training and testing in two English prisons, was undertaken in an attempt to ensure relevance [16].

Many articles did not include information about the wording of the offer, meaning reviewers were forced to assume that testing really was, for the most part, offered as opt-out [37–41, 46, 53, 55, 67, 77]. It is important that details, preferably a standard quote, for the process of gaining consent to test, are presented, providing transparency and allowing for an assessment of the true nature of the offer process [7, 8, 42, 43, 48].

The reviewers were also heavily reliant on author justifications for failure to offer testing and prisoner opt-out. Qualitative insights into the reasoning processes of different prison stakeholders were scarce and therefore often inferred. In all reviews that utilise realist synthesis methodology, there will be some judgement involved when making inferences between the data found in included studies [18, 78], however further qualitative research is required to provide greater insight into the decision-making process of relevant stakeholders.

The framework sketched out in this review is therefore intended to provide a starting point on which to build our understanding of opt-out BBV programmes in prison [71]. The CMOcs presented are falsifiable and require further refinement using primary data [71]. To help enable this process, and in line with best practice, the authors have attempted to maximise the transparency of the review process [78].

## Conclusion

Opt-out testing programmes for BBVs within prison have been found to increase test uptake, however evaluative work has reported a high degree of variability in key outcomes. This synthesis highlighted important implementation considerations, which influence the effectiveness of these programmes. The fidelity of opt-out was also questionable, both at the programme level and because of inter and intra health worker variability in the way testing is offered.

Programme implementers are encouraged to utilise Nudge Theory within their opt-out BBV test design, to take full advantage of the Default Effect for public health benefit. They are also encouraged to think carefully about the timing of the test offer, work with prison authorities to overcome logistical barriers to accessing prisoners, explore ways of enhancing the fidelity of an opt-out offer, and ensure the realisation of key programme resources that can mitigate testing costs.

## Additional files

Additional file 1: Search strategy (MEDLINE). An example search strategy used during phase 2. (DOCX 14 kb)

Additional file 2: Results from unstructured search. Articles used in the development of a provisional programme theory during phase 1. (DOCX 17 kb)

Additional file 3: Result from additional unstructured searches. Articles used to reinforce programme theory during phase 3. (DOCX 22 kb)

Additional file 4: Generic process for opt-out. A diagram detailing the generic steps involved in the opt-out BBV test programme, implemented throughout English prisons. (DOCX 25 kb)

Additional file 5: CMOcs. A table detailing the full list of CMOcs developed during the conduct of the rapid-realist review. (DOCX 227 kb)

Additional file 6: Wording for opt-out offer. A script developed to help guide frontline health staff deliver an opt-out BBV test. Wording shared with NHS England commissioners. (DOCX 15 kb)

## Abbreviations

BBV: Blood-borne virus
CMO: Context-mechanism-outcome
CMOc: Context-mechanism-outcome configuration
DBST: Dried blood spot testing
HBV: Hepatitis B
HCV: Hepatitis C
HIV: Human immunodeficiency virus
HMPPS: Her Majesty’s Prison and Probation Service
NHSE: National Health Service England
PHE: Public Health England
RRR: Rapid-realist review
