# Supplementary material for: Understanding how, why, for whom, and under what circumstances opt-out blood-borne virus testing programmes work to increase test engagement and uptake within prison: a rapid-realist review
Source: BMC Health Serv Res. 2019 Mar 8;19:152. doi: 10.1186/s12913-019-3970-z (PMC6408812; doi:10.1186/s12913-019-3970-z)
Supplement: Supplementary file 1 — Search strategy (MEDLINE). An example search strategy used during phase 2. (DOCX 14 kb) [file 12913_2019_3970_MOESM1_ESM.docx]

## Additional file 1: Search strategy (MEDLINE)

Database: Ovid MEDLINE(R) Epub Ahead of Print, In-Process & Other Non-Indexed Citations, Ovid MEDLINE(R) Daily and Ovid MEDLINE(R) <1946 to Present>

Search Strategy:

--------------------------------------------------------------------------------

1 Prisoners/ (15231)

2 offender*.mp. [mp=title, abstract, original title, name of substance word, subject heading word, keyword heading word, protocol supplementary concept word, rare disease supplementary concept word, unique identifier, synonyms] (10327)

3 prisoner*.mp. [mp=title, abstract, original title, name of substance word, subject heading word, keyword heading word, protocol supplementary concept word, rare disease supplementary concept word, unique identifier, synonyms] (18395)

4 convict*.mp. [mp=title, abstract, original title, name of substance word, subject heading word, keyword heading word, protocol supplementary concept word, rare disease supplementary concept word, unique identifier, synonyms] (5961)

5 detainee*.mp. [mp=title, abstract, original title, name of substance word, subject heading word, keyword heading word, protocol supplementary concept word, rare disease supplementary concept word, unique identifier, synonyms] (832)

6 inmate*.mp. [mp=title, abstract, original title, name of substance word, subject heading word, keyword heading word, protocol supplementary concept word, rare disease supplementary concept word, unique identifier, synonyms] (4702)

7 incarcerated.mp. [mp=title, abstract, original title, name of substance word, subject heading word, keyword heading word, protocol supplementary concept word, rare disease supplementary concept word, unique identifier, synonyms] (5864)

8 1 or 2 or 3 or 4 or 5 or 6 or 7 (36284)

9 Prisons/ (8672)

10 prison*.mp. [mp=title, abstract, original title, name of substance word, subject heading word, keyword heading word, protocol supplementary concept word, rare disease supplementary concept word, unique identifier, synonyms] (26108)

11 gaol*.mp. [mp=title, abstract, original title, name of substance word, subject heading word, keyword heading word, protocol supplementary concept word, rare disease supplementary concept word, unique identifier, synonyms] (93)

12 jail*.mp. [mp=title, abstract, original title, name of substance word, subject heading word, keyword heading word, protocol supplementary concept word, rare disease supplementary concept word, unique identifier, synonyms] (2864)

13 ((Correction* or penal or remand* or detention or custody) adj2 (centre or department or facility* or system*)).mp. [mp=title, abstract, original title, name of substance word, subject heading word, keyword heading word, protocol supplementary concept word, rare disease supplementary concept word, unique identifier, synonyms] (1989)

14 Penitent*.mp. [mp=title, abstract, original title, name of substance word, subject heading word, keyword heading word, protocol supplementary concept word, rare disease supplementary concept word, unique identifier, synonyms] (601)

15 9 or 10 or 11 or 12 or 13 or 14 (28408)

16 8 or 15 (44020)

17 mass screening/ or mandatory testing/ (94669)

18 Diagnosis/ (17347)

19 "Diagnostic Techniques and Procedures"/ (2880)

20 (Mandatory adj (test* or screen* or diagnos* or identif* or assess*)).mp. [mp=title, abstract, original title, name of substance word, subject heading word, keyword heading word, protocol supplementary concept word, rare disease supplementary concept word, unique identifier, synonyms] (1349)

21 (Systematic* adj (test* or screen* or diagnos* or identif* or assess*)).mp. [mp=title, abstract, original title, name of substance word, subject heading word, keyword heading word, protocol supplementary concept word, rare disease supplementary concept word, unique identifier, synonyms] (11619)

22 (Routine adj (test* or screen* or diagnos* or identif* or assess*)).mp. [mp=title, abstract, original title, name of substance word, subject heading word, keyword heading word, protocol supplementary concept word, rare disease supplementary concept word, unique identifier, synonyms] (18344)

23 (Compulsory adj (test* or screen* or diagnos* or identif* or assess*)).mp. [mp=title, abstract, original title, name of substance word, subject heading word, keyword heading word, protocol supplementary concept word, rare disease supplementary concept word, unique identifier, synonyms] (121)

24 (Obligatory adj (test* or screen* or diagnos* or identif* or assess*)).mp. [mp=title, abstract, original title, name of substance word, subject heading word, keyword heading word, protocol supplementary concept word, rare disease supplementary concept word, unique identifier, synonyms] (87)

25 opt-out.mp. [mp=title, abstract, original title, name of substance word, subject heading word, keyword heading word, protocol supplementary concept word, rare disease supplementary concept word, unique identifier, synonyms] (1191)

26 opt* out.mp. [mp=title, abstract, original title, name of substance word, subject heading word, keyword heading word, protocol supplementary concept word, rare disease supplementary concept word, unique identifier, synonyms] (1561)

27 17 or 18 or 19 or 20 or 21 or 22 or 23 or 24 or 25 or 26 (144087)

28 16 and 27 (934)

29 limit 28 to yr="2000 - 2017" (662)

***************************
