# Supplementary material for: Understanding how, why, for whom, and under what circumstances opt-out blood-borne virus testing programmes work to increase test engagement and uptake within prison: a rapid-realist review
Source: BMC Health Serv Res. 2019 Mar 8;19:152. doi: 10.1186/s12913-019-3970-z (PMC6408812; doi:10.1186/s12913-019-3970-z)
Supplement: Supplementary file 2 — Results from unstructured search. Articles used in the development of a provisional programme theory during phase 1. (DOCX 17 kb) [file 12913_2019_3970_MOESM2_ESM.docx]

| **First author, year** | **Title** | **Country** | **Format** | **Aims** |
| --- | --- | --- | --- | --- |
| Public Health England (2014) | BBV Bulletin: Issue 2, July 2014 | UK | Blood Borne Virus Bulletin | Provide a monthly update report covering the introduction of opt-out blood borne virus testing in prisons from Public Health England and NHS England. |
| Public Health England (2014) | BBV Bulletin: Issue 3, September 2014 | UK | Blood Borne Virus Bulletin | Provide a monthly update report covering the introduction of opt-out blood borne virus testing in prisons from Public Health England and NHS England. |
| Offender Health Research Network (2008) | An evaluation of the reception screening process used in England/Wales | UK | Report | To evaluate the standard reception screening tool and present views from a range of stakeholders regarding reception screening, including challenges to practice and suggestions for improvement. |
| Public Health England (2014) | Blood borne virus testing flash cards | UK | Clinical guidance materials | To provide basic guidance for clinicians implementing opt-out blood borne virus testing in the prison system. |
| Public Health England (2014) | Opt-out blood borne virus test algorithm | UK | Guidance materials | Details testing algorithm for blood borne viruses. |
| Public Health England (2014) | Opt-out blood borne virus test algorithm guidance notes | UK | Guidance materials | To provide guidance on the opt-out test algorithm for blood borne viruses in the English prison system. |
| Public Health England (2014) | Frequently asked questions to support the opt-out testing policy | UK | Guidance materials | To provide standard answers for frequently asked questions concerning opt-out testing for blood borne viruses within prisons. |
| The Hepatitis C Trust (2016) | Hepatitis C prevention, diagnosis, and treatment in prisons in England | UK | Report/guidance materials | To provide commissioners and prison healthcare teams with practical guidance regarding the implementation of opt-out blood borne virus testing and related hepatitis C care pathways. |
| The Hepatitis C Trust (2016) | The blood-borne virus opt-out testing policy for prisons in England: An analysis of need towards full implementation | UK | Report | Highlight practical advice from different prisons offering opt-out BBV testing. |
| REACH (2015) | Prison BBV Champions Training | UK | Slides and note book | The training booklet provided to prison healthcare to support the service reconfiguration to opt-out blood-borne virus testing. |
| Public Health England (2016) | BBV opt-out testing in Prisons – A London update | UK | Presentation on evaluation | Summarise the rationale of opt-out BBV testing in prisons and provide data on current testing rates for the various London prisons. Also provides recommendations informed by national Phase 1 pathfinder evaluation. |
| Leidel (2016) | A comprehensive theoretical framework for the implementation and evaluation of opt-out HIV testing | Australia | Journal article | To consider the application of three theories to the implementation and evaluation of an opt-out HIV testing programme: Behavioural Economics, the Health Belief Model, and Normalisation Process Theory. |
| Johnson (2004) | Defaults and donation decisions | U.S. | Journal article | Review evidence that suggests the preference to become a donor is not well formed and that the donation decision in constructed in response to the question. |
| Bronchetti (2011) | When a nudge isn’t enough: Defaults and saving among low-income tax filers | U.S. | NBER Working Paper | Present a field experiment that evaluates the effect of defaults on saving decisions among low-income tax filers. |
| Johnson (2000) | Defaults, framing and privacy: Why opting in-opting out | U.S. | Journal article | Explore the issue of difference in opt-in and opt-out responses in light of current public debate concerning online privacy and permission for marketing. |
| Keller (2011) | Enhanced active choice: A new method to motivate behaviour change | U.S. | Journal article | Present a series of studies that demonstrate the effectiveness of an alternative to opt-out (active choice), where there is no default, but decision makers are required to make a choice. |
| Halpern (2007) | Harnessing the power of default options to improve health care | U.K. | Journal article | Discuss the role of defaults in healthcare. |
| Sunstein (2008) | Nudge: Improving decision making about health, wealth and happiness | U.S. | Book | Applies behavioural economic “Nudge” theory to a range of social problems. |
| Montoy (2016) | Patient choice in opt-in, active choice and opt-out HIV screening: randomized clinical trial | U.S. | Journal article | To explore the effect of default test offers – opt-in, opt-out, and active choice, on acceptance of HIV testing in an emergency department. |
| Bellman (2001) | To opt-in or opt-out? It depends on the question | U.S. | Journal article | Systematically explore the influence of question framing and response defaults on consumers’ privacy preference. |
