# Supplementary material for: Understanding how, why, for whom, and under what circumstances opt-out blood-borne virus testing programmes work to increase test engagement and uptake within prison: a rapid-realist review
Source: BMC Health Serv Res. 2019 Mar 8;19:152. doi: 10.1186/s12913-019-3970-z (PMC6408812; doi:10.1186/s12913-019-3970-z)
Supplement: Supplementary file 3 — Result from additional unstructured searches. Articles used to reinforce programme theory during phase 3. (DOCX 22 kb) [file 12913_2019_3970_MOESM3_ESM.docx]

| **First author, year** | **Title** | **Country** | **Disease** | **Population** | **Study design** | **Justification for inclusion** |
| --- | --- | --- | --- | --- | --- | --- |
| Khaw, 2007 | “I just keep thinking I haven’t got it because I’m not yellow’: a qualitative study of the factors that influence the uptake of Hepatitis C testing by prisoners. | U.K. | HCV | Patients in prison | Qualitative interview | Article provided information on patient justification for not undertaking hepatitis C testing within a prison context. Data used to reinforce several CMO configurations. |
| Rhodes, 2008 | The social production of hepatitis C risk among injecting drug users: a qualitative analysis. | Mixed | HCV | Injecting drug users | Meta-ethnography | Although not prison specific, the article provided interesting insight into injecting drug users’ perceptions of hepatitis C and HIV and how risk perceptions shaped considerations of treatment. Information used to reinforce thinking around risk perception related CMOs. |
| Strauss, 2008 | Barriers and facilitators to undergoing hepatitis C virus testing through drug treatment programs. | U.S. | HCV | Patients attending drug treatment programs | Qualitative interview | Article provided reasons for testing/not testing that were used to validate a number of CMO configurations. Article also highlighted similarities in justification for either testing or not testing between HCV and HIV. |
| Harris, 2016 | Finding the undiagnosed: a qualitative exploration of hepatitis C diagnosis delay in the United Kingdom. | U.K. | HCV | People living with HCV | Qualitative interview/focus group | Aimed to explore the context of a diagnosis delay among people living with HCV in the UK. Article provided explanations for why individuals with HCV may avoid testing. Was used to strengthen thinking around risk perception CMO and prioritisation. |
| The Hepatitis C Trust, 2012 | Increasing hepatitis B and C testing in the prison setting: the use of new diagnostics at HMP Manchester. | U.K. | HCV/HBV | Patients in prison | Report | Article provided information on barriers to testing within the prison setting that was used to validate CMOs surrounding fear of needles and stigma. |
| Young, 2009 | Opt-out testing for stigmatised diseases: a social psychological approach to understanding the potential effect of recommendations for routine HIV testing. | U.S. | HIV | General population | Psychological experiment | Provided information on testing for a stigmatised disease. Articles idea about normative position and counter normative behaviour helped validate CMO related to stigma-based testing. |
| Noland, 2015 | Understanding patients’ perspectives on opt-out, incentivised and mandatory HIV testing. | U.S. | HIV | Sero-positive and negative patients | Qualitative interviews | Article discussed the theory of opt-out testing for HIV. Helped validate a range of CMOs as well as contribute ideas about the impact of the default effect during opt-out testing. |
| Sabharwal, 2010 | Jail-based providers’ perceptions of challenges to routine HIV testing in New York City Jails. | U.S. | HIV | Patients in prison | Mixed Methods | Article provided rare insight into health worker perceptions of running a routine HIV testing service within high-turnover jails. Although the service was not opt-out, due to a legal requirement for written consent, the data on context and mechanism was used to reinforce a number of programme theories. |
| MacDonald, 2006 | People with problematic drug use and HIV/AIDS in European prisons: an issue of patient confidentiality | Mixed | Mixed | Prison staff, medical staff, and patients in prison | Interviews | Provides broad insight into issues of confidentiality throughout EU prisons in terms of the diagnosis and treatment of potentially stigmatising medical conditions. Data was used to reinforce theories related to confidentiality. |
| Hickman, 2007 | Increasing the uptake of hepatitis C virus testing among injecting drug users in specialist drug treatment and prison settings by using dried blood spots for diagnostic testing: a cluster randomised controlled trial | U.K. | HCV | Specialist Drug Treatment and Prison | Cluster randomised control trial | Article provides quantitative evidence regarding method of sample acquisition and test uptake. Used to reinforce theories related to fear of an invasive test method. |
| Craine, 2015 | A stepped wedge cluster randomized control trial of dried blood spot testing to improve the uptake of hepatitis C antibody testing within UK prisons | U.K. | HCV | Prison | Stepped wedge cluster randomised control trial | Article provides quantitative evidence regarding method of sample acquisition and test uptake. Used to reinforce theories related to fear of an invasive test method. |
