# Supplementary material for: Understanding how, why, for whom, and under what circumstances opt-out blood-borne virus testing programmes work to increase test engagement and uptake within prison: a rapid-realist review
Source: BMC Health Serv Res. 2019 Mar 8;19:152. doi: 10.1186/s12913-019-3970-z (PMC6408812; doi:10.1186/s12913-019-3970-z)
Supplement: Supplementary file 4 — Generic process for opt-out. A diagram detailing the generic steps involved in the opt-out BBV test programme, implemented throughout English prisons. (DOCX 25 kb) [file 12913_2019_3970_MOESM4_ESM.docx]

## Additional file 4: Generic process for opt-out

Pre-test educational activity

Blood sample taken and prepared for transportation to lab

Lab processes test and returns results to prison

BBV results delivered and post-test discussion conducted

Verbal opt-out

Reason recorded

Patient provided ongoing opt-out offers and harm minimisation

Eligible patients engaged for testing within 72 hours of reception at the prison

Opt-out offer
