# Supplementary material for: Understanding how, why, for whom, and under what circumstances opt-out blood-borne virus testing programmes work to increase test engagement and uptake within prison: a rapid-realist review
Source: BMC Health Serv Res. 2019 Mar 8;19:152. doi: 10.1186/s12913-019-3970-z (PMC6408812; doi:10.1186/s12913-019-3970-z)
Supplement: Supplementary file 5 — CMOcs. A table detailing the full list of CMOcs developed during the conduct of the rapid-realist review. (DOCX 227 kb) [file 12913_2019_3970_MOESM5_ESM.docx]

## Engagement (offer) related CMOs

| Theory | Context | Mechanism -----------------------------------------------> | | = Proximal outcome | Supporting references |
| --- | --- | --- | --- | --- | --- |
|  |  | **+ Resource** | **Response** |  |  |
| Delayed test offer | *Prison with a high population turnover* | *Programme mandated delay in test offer* | *N/A* | *Proportion of prisoners not there to be offered a test* | (Kavasery, Maru, Sylla, et al. 2009; Public Health England 2015; Rice 2010; Lucas et al. 2016; CDC 2009; Peter 2009; Beckwith et al. 2010; Cole et al. 2014; Beckwith et al. 2012; Centers for Disease Control and Prevention 2010; Kavasery, Maru, Cornman-Homonoff, et al. 2009; Nelwan et al. 2016; Rumble et al. 2015; Sabharwal et al. 2010). |
| Early testing and capacity to consent | *High proportion of prisoners lack capacity to consent on the first night* | *Health worker recognises inability to consent* | *Views inability to consent as important* | *Prisoner not offered a test (see re-booking theory)* | (Centres for Disease Control and Prevention 2009; Jackie Walker et al. 2005; Centers for Disease Control and Prevention 2013; Kavasery, Maru, Cornman-Homonoff, et al. 2009; Kavasery, Maru, Sylla, et al. 2009; Sabharwal et al. 2010). |
|  |  | *Health worker fails to recognise inability to consent* | *Health worker believes it is okay to proceed* | *Prisoner offered a test, risking test uptake without informed consent.* |  |
| Language barriers | *Certain prisons, either in metropolitan areas or designated as immigration centres, have a high proportion of foreign nationals, complicating communication between health workers and prisoners* | *Translation service available* | *Health worker able to offer testing* | *Prisoner offered a test* | (Rumble et al. 2015; Sabharwal et al. 2010)**.** |
|  |  | *Translation service not available* | *Health worker unable to offer testing* | *Prisoner not offered a test (see re-booking theory)* |  |
| Prioritisation of security | *Prison officers have a challenging role and budget cuts have left them short staffed* | *Requirement of officers to bring prisoners to clinic* | *Prioritisation of security processes over transportation* | *Proportion of prisoners do not arrive at clinic to be offered a test (see re-booking theory)* | (Rice 2010; Schoenbachler et al. 2016; Peter 2009; Cole et al. 2014; Jack et al. 2017; Sabharwal et al. 2010). |
|  |  | *Prisoners free to move to clinic unaccompanied (“free-flow”)* | *Prisoners that wish to attend the clinic are not reliant on anyone else to supervise them* | *Those prisoners that wish to attend the clinic, arrive to be offered a test* |  |
| Institutional scramble | *Access to a prisoner is often reduced, once they are moved from the induction wing, as they are harder to physically locate within the wider prison population. This encourages an institutional scramble in the first few days of incarceration, with NGO’s, Chaplaincy, Immigration, Courts and Healthcare all vying to engage prisoners.* | *Requirement to engage prisoner for testing early* | *N/A* | *Proportion of prisoners not accessible, as being seen by another organisation, and therefore not offered a test (see re-booking theory)* | (Rice 2010; Schoenbachler et al. 2016; Peter 2009; Cole et al. 2014). |

| Theory | Context | Mechanism -----------------------------------------------> | | = Proximal outcome | Supporting references |
| --- | --- | --- | --- | --- | --- |
|  |  | **+ Resource** | **Response** |  |  |
| Refusal to attend clinic | *Testing conducted concurrently with other prison activities* | *Attendance at clinic becomes an opportunity cost for the prisoner* | *Prisoner prioritises healthcare over other activity* | *Prisoner agrees to transportation to clinic (see prioritisation theory)* | (Rice 2010; Peter 2009; Rumble et al. 2015). |
|  |  |  | *Prisoner prioritises other activity over healthcare* | *Prisoner disagrees to transportation to clinic* |  |
| Provider capacity to run clinics | *Prisons are a demanding place to work (high burden of mental illness, physical morbidity, and regular medical emergencies) and budget deficits result in health staff cuts* | *Capacity of health staff stretched* | *Prioritisation of urgent conditions and emergencies* | *Testing clinic delayed or cancelled, and prisoners require re-booking (see rebooking prisoners theory)* | (Public Health England 2015; Rice 2010; Grinstead et al. 2003; Peter 2009; Cole et al. 2014; Nelwan et al. 2016; Sabharwal et al. 2010). |
| Rebooking prisoners |  | *Missed prisoners need to be re-booked by health worker* | *Prioritise medical emergencies and conducting other tasks that require immediate attention* | *Test offer delayed (see delayed test offer theory)* | (Public Health England 2015; Rice 2010; Grinstead et al. 2003; Peter 2009; Cole et al. 2014; Nelwan et al. 2016; Sabharwal et al. 2010). |

## Pre-test education

| Theory | Context | Mechanism -----------------------------------------------> | | = Proximal outcome | Supporting references |
| --- | --- | --- | --- | --- | --- |
|  |  | **+ Resource** | **Response** |  |  |
| Personal interpretation of risk | *Misconceptions around BBVs are common amongst prisoners* | *Educational information about modes of transmission and symptoms of disease* | *Re-assess risk of infection in light of new information* | *Prisoner able to more accurately interpret their risk of infection (see personal interpretation of risk T2)* | (Rosen 2007; Muessig et al. 2016; Grodensky et al. 2016; Rumble et al. 2015; Nelwan et al. 2016; Khaw et al. 2007; Sabharwal et al. 2010).  (Kavasery, Maru, Sylla, et al. 2009; Rice 2010; Spaulding et al. 2015; Cole et al. 2014; Public Health England 2016; Centers for Disease Control and Prevention 2013; Kavasery, Maru, Cornman-Homonoff, et al. 2009; Rumble et al. 2015; Nelwan et al. 2016). |

## Opt-out offer

| Theory | Context | Mechanism -----------------------------------------------> | | = Proximal outcome | Supporting references |
| --- | --- | --- | --- | --- | --- |
|  |  | **+ Resource** | **Response** |  |  |
| Opt-out fidelity T1 | *Programme implementers misinterpret how to deliver an opt-out test* | *Training provided to health workers encourage opt-in or mandatory test offer* | *Health workers encouraged to deliver a poor opt-out message* | *Opt-in or mandatory test offer* | (Public Health England 2015; Rosen et al. 2015; Grodensky et al. 2016; Wohl et al. 2010; Public Health England 2016). |
| Opt-out fidelity T2 | *Opt-out testing not the norm* | *Little training and no standard script* | *Health worker misinterprets how to deliver test offer* | *Opt-in or mandatory test offer* |  |
|  |  | *Training and/or script provided* | *Health worker understands how to deliver the test offer* | *Opt-out test offer* |  |
| Opt-out fidelity T3 | *Health worker fatigued, distracted, or has a high level of rapport with a prisoner* | *No standard script* | *Health worker defaults to old way of offering a test* | *Opt-in test offer* |  |
| Language barriers T3 | *Language barrier between prisoner and health worker, but translation service present* | *Failure to highlight the need for testing to be opt-out to translation provider* | *Misinterpretation of how to offer testing by translation provider* | *Opt-in or mandatory test offer* | (Rumble et al. 2015; Sabharwal et al. 2010)**.** |

## Test uptake

| Theory | Context | Mechanism -----------------------------------------------> | | = Proximal outcome | Supporting references |
| --- | --- | --- | --- | --- | --- |
|  |  | **+ Resource** | **Response** |  |  |
| Personal interpretation of risk T1 | *Prisoner views themselves as at high risk of infection* | *Testing presented as an opportunity to confirm serostatus and supportive information provided* | *Testing perceived to be an opportunity to confront infection or be reassured by a negative result* | *Encourage test uptake* | (Rosen 2007; Muessig et al. 2016; Grodensky et al. 2016; Rumble et al. 2015; Nelwan et al. 2016; Khaw et al. 2007; Sabharwal et al. 2010).  (Kavasery, Maru, Sylla, et al. 2009; Rice 2010; Spaulding et al. 2015; Cole et al. 2014; Public Health England 2016; Centers for Disease Control and Prevention 2013; Kavasery, Maru, Cornman-Homonoff, et al. 2009; Rumble et al. 2015; Nelwan et al. 2016). |
|  |  | *Testing presented as an opportunity to confirm serostatus and supportive information not provided* | *Prisoner feels unable to cope with a positive result* | *Encourage opt-out* |  |
| Personal interpretation of risk T2 | *Prisoner views themselves as at low risk of infection* | *Test programme reduces other barriers to testing* | *Prisoner seeks peace of mind* | *Encourage test uptake* |  |
|  |  | *Other barriers to testing present* | *Testing viewed as an unnecessary burden* | *Encourage opt-out* |  |

| Theory | Context | Mechanism -----------------------------------------------> | | = Proximal outcome | Supporting references |
| --- | --- | --- | --- | --- | --- |
|  |  | **+ Resource** | **Response** |  |  |
| Confidentiality and stigma | *BBVs stigmatised within a prison context* | *Confidentiality maintained inside and outside of clinic* | *Prisoner feels safe to engage with testing and share personal information* | *Test uptake encouraged* | (Kavasery, Maru, Sylla, et al. 2009; Elkington et al. 2016; Spaulding et al. 2015; Lucas et al. 2016; Grinstead et al. 2003; CDC 2009; Muessig et al. 2016; J. Walker et al. 2005; Beckwith et al. 2010; Jack et al. 2017; Beckwith et al. 2012; Kavasery, Maru, Cornman-Homonoff, et al. 2009; Khaw et al. 2007; Sabharwal et al. 2010; MacDonald 2006). |
|  |  | *Poor confidentiality maintenance inside and outside of clinic* | *Prisoner distrusts prison healthcare’s ability to maintain confidentiality and fears stigma as a result of engagement* | *Opt-out encouraged* |  |
| Previous experience of poor quality of care | *Prisoners experienced (directly/indirectly) poor quality of care* | *No attempt to build rapport and put prisoner at ease* | *Testing viewed as an unpleasant or dangerous experience* | *Encourage opt-out* | (Elkington et al. 2016; J. Walker et al. 2005). |
|  |  | *Professional approach, attempts to build rapport, and provide opportunities for prisoners to verbalise concerns related to engaging with healthcare* | *Reassured about engaging with healthcare* | *Encourage test uptake* |  |
| Fear consequences of opt-out | *Prisons are inherently coercive environments where a prisoner has little self-determination* | *Prisoners not informed that they will not be reprimanded in the event of opt-out* | *Prisoner fears consequences of opt-out* | *Comply with testing even if they do not wish to test* | (Rosen et al. 2015; Centres for Disease Control and Prevention 2009; Grodensky et al. 2016; Wohl et al. 2010; Kavasery, Maru, Cornman-Homonoff, et al. 2009). |
| Institutional recommendations and trust T1 | *Prisoner trusts the prison staff and healthcare providers* | *Institutional social pressure* | *Encouraged to test* | *Encourage test uptake* | (Rice 2010; Peter 2009; Elkington et al. 2016; Grinstead et al. 2003). |
| Institutional recommendations and trust T2 | *Prisoner distrusts prison staff and healthcare providers* |  | *Viewed as a coercive process of surveillance* | *Encourage opt-out* |  |
| Resistance to taking on prisoner status | *The intake process is a system of induction designed to transition a free citizen into a prisoner of the state. Prisoners anticipating a short period of incarceration* | *Testing incorporated into intake procedures* | *Resist induction procedures (including testing) to avoid taking on the status of a prisoner* | *Encourage opt-out* | (Kavasery, Maru, Sylla, et al. 2009; Kavasery, Maru, Cornman-Homonoff, et al. 2009; Gagnon et al. 2013). |
| Refusal to engage in protest | *New intake into a prison often have grievances with police from the arrest and are angry at the court’s decision to incarcerate them, particularly if this is seen to be unfair and has implications for family or other social networks.* | *Testing requires collaboration between prisoner and health staff* | *Frustration and anger manifests in complete refusal to engage as a form of protest* | *Encourage opt-out* | (Rumble et al. 2015; Sabharwal et al. 2010; Jack et al. 2017)**.** |

## Macro-to-micro mechanism

| Theory | Interaction between contextual features | Mechanism -----------------------------------------------> | = Conditioning of prisoner decision making | Supporting references |
| --- | --- | --- | --- | --- |
| Counter-normative behaviour T1 | *In a high-test uptake but stigmatised setting, where confidentiality is poorly maintained* | *Activities that are counter-normative may signal that a prisoner has something to hide* | *Prisoner encouraged to comply with norm and take a test (even if they do not wish to)* | (Young et al. 2009; Noland et al. 2015; Kavasery, Maru, Sylla, et al. 2009; Elkington et al. 2016; Muessig et al. 2016). |
| Counter-normative behaviour T2 | *In a low-test uptake but stigmatised setting, where confidentiality is poorly maintained* |  | *Prisoner encouraged to comply with norm and opt-out (even if they do not wish to)* |  |
| Situational group pressure T1 | *Testing procedures conducted sequentially on a group that are able to communicate and become supportive of testing* | *Autonomy of individual eroded by situational group pressures* | *Peer pressure encourages test uptake (even if individual does not wish to)* | (Grodensky et al. 2016; Gagnon et al. 2013). |
| Situational group pressure T2 | *Testing procedures conducted sequentially on a group that are able to communicate and become oppositional to testing* |  | *Peer pressure encourages opt-out (even if individual does not wish to)* |  |

| Theory | Context | Mechanism -----------------------------------------------> | | = Proximal outcome | Supporting references |
| --- | --- | --- | --- | --- | --- |
|  |  | **+ Resource** | **Response** |  |  |
| Coping with a positive diagnosis | *BBVs are a situational concern for many people in prison* | *Supportive information (e.g. treatment options, dispelling myths around prognosis, and details of available psycho-social support) provided* | *Prisoner reassured about their ability to cope if they test positive* | *Encouraging test uptake* | (Rhodes & Treloar 2008; Kavasery, Maru, Sylla, et al. 2009; Elkington et al. 2016; Rosen 2007; Grinstead et al. 2003; J. Walker et al. 2005; Rumble et al. 2015; Khaw et al. 2007). |
|  |  | *Supportive information not provided* | *Prisoner feels unable to cope with the perceived burden associated with a positive result* | *Encourage opt-out* |  |
| Fear of invasive procedure | *A proportion of prisoner’s fear needles* | *Testing conducted using a venous sample method* | *Prisoners that are uncomfortable with the method of acquisition* | *Encourage opt-out* | (Beckwith et al. 2011; Grinstead et al. 2003; Muessig et al. 2016; Kavasery, Maru, Cornman-Homonoff, et al. 2009; Nelwan et al. 2016; Rumble et al. 2015; Hickman et al. 2008; Craine et al. 2015). |
|  |  | *Testing conducted using a less intrusive sample method (oral/dried blood spot)* | *Prisoners that fear needles* | *Not encouraged to opt-out* |  |

## Micro-to-macro mechanism

| Theory | Programme outcome | Mechanism -----------------------------------------------> | = Contextual transformation | Supporting references |
| --- | --- | --- | --- | --- |
| Routine offer leads to offer normalisation | *Opt-out programme successfully offers most prisoners a test* | *Being offered a test is the norm experience* | *Being offered a test becomes unexceptional* | (Beckwith et al. 2011; Public Health England 2015; Rosen et al. 2016; J. Walker et al. 2005; Kavasery, Maru, Sylla, et al. 2009; Centers for Disease Control and Prevention 2011). |
