# Supplementary material for: Understanding how, why, for whom, and under what circumstances opt-out blood-borne virus testing programmes work to increase test engagement and uptake within prison: a rapid-realist review
Source: BMC Health Serv Res. 2019 Mar 8;19:152. doi: 10.1186/s12913-019-3970-z (PMC6408812; doi:10.1186/s12913-019-3970-z)
Supplement: Supplementary file 6 — Wording for opt-out offer. A script developed to help guide frontline health staff deliver an opt-out BBV test. Wording shared with NHS England commissioners. (DOCX 15 kb) [file 12913_2019_3970_MOESM6_ESM.docx]

## Additional file 6: Wording for opt-out offer

“We screen everybody entering this prison for hepatitis B, hepatitis C, and HIV. Screening is free, confidential and the sample will not be used for anything other than this test. You can be infected and still feel healthy, so it is important to test, even if you feel fit and well. If you have hepatitis C, we can treat you with new medication that works in almost all cases, usually with no side effects. Are you happy to proceed?”.

### Programme theory behind consent acquisition wording

1. A test offer where a patient is asked whether they would like to test, fails to align the default with the public health objective, limiting test uptake.
2. In a punitive environment, a test offer where the patient is not told they can decline, is frequently interpreted by patients as mandatory, increasing test uptake but without consent.
3. In an environment where the patient has few chances to express agency, a test offer that explicitly highlights the patients right to decline the test (e.g. “we test everyone unless you refuse”), invites patients to take this rare opportunity to express agency and opt-out.
4. In a punitive environment, where patients have little chance to express agency, asking patients if they are happy to proceed with testing, passively acquires patient consent, without limiting test uptake.
